# Development and application of a prosthetist-specific rectification template based on artificial intelligence for the fabrication of transfemoral prosthetic sockets

**DOI:** 10.1371/journal.pone.0356483

**Published:** 2026-08-19

**Authors:** Maria Grazia Santi, Stefania Fatone, Andrew H. Hansen, Steven A. Gard, Andrea Giovanni Cutti, Residual Limb Shape Capture Group

**Affiliations:** 1 INAIL, Vigorso di Budrio, Bologna, Italy; 2 Department of Industrial Engineering, University of Padova, Padova, Province of Padua, Italy; 3 Department of Rehabilitation Medicine, University of Washington, Seattle, Washington, United States of America; 4 Minneapolis VA Health Care System, Minneapolis, Minnesota, United States of America; 5 Department of Family Medicine and Community Health, Department of Biomedical Engineering, University of Minnesota, Minneapolis, Minnesota, United States of America; 6 Northwestern University Prosthetics-Orthotics Center, Department of Physical Medicine and Rehabilitation, Feinberg School of Medicine, Chicago, Illinois, United States of America; 7 Jesse Brown VA Medical Center, Chicago, Illinois, United States of America; St Mary's University, UNITED STATES OF AMERICA

## Abstract

The prosthetic socket is the most critical component of a lower limb prosthesis, requiring precise customization to the individual’s residual limb. This study presents proof-of-concept for a novel artificial intelligence (AI)-driven rectification template for transfemoral sockets tailored to a single prosthetist. Using a dataset of nine persons with transfemoral amputation, the study workflow required the manual casting and 3D scanning of unrectified and rectified plaster positives, anatomical landmark identification, and unsupervised training of an algorithm using Principal Component Analysis (PCA). The AI captured both explicit and implicit rectification strategies, generating an average rectification template and synergistic modes of variation. Validation was conducted via a leave-one-out approach, comparing AI-generated versus manually crafted rectified positives using clinically-relevant metrics: perimeter and volume differences. The first four PCA modes explained 78% of rectification variability, with key modifications observed in distal and medial regions. Volume differences between AI and manually rectified positives were within clinically acceptable limits for all participants, with 44% rated “good” and 56% “acceptable”. This proof-of-concept provides a new perspective on the application of AI to replicate prosthetist-specific rectification strategies for transfemoral sockets. It may potentially help streamline fabrication by capturing both explicit and implicit prosthetist knowledge. The approach may be useful in clinical training, documentation, and socket fabrication, particularly in resource-limited settings, and may contribute to more consistent and efficient prosthetics care.

## Introduction

The socket is the most important component of a lower limb prosthesis. While all other components are mass-produced, the socket is a person-specific element, fabricated by prosthetists based on characteristics of the individual [1–5]. The socket is usually handcrafted from a negative mould of the individual’s residual limb, typically made by wrapping wet plaster or fiberglass bandages around the limb and allowing them to harden. A positive mould is then made by filling the negative mould with liquid plaster, allowing it to harden, and then stripping away the plaster or fiberglass bandages. This initial positive mould, also known as an “unrectified positive” (UP), is then shaped by the prosthetist using a process called “rectification”, in which material is added or removed in specific anatomical areas corresponding to the chosen socket design. The result is a “rectified positive” (RP) that has the shape of the socket interface with the residual limb [6–11]. From this shape, the socket is then manufactured using either composite lamination, vacuum forming of a thermoplastic material, 3D printing, or a combination thereof. The shape and volume of the socket are then fine-tuned with the prosthesis user by static evaluation and, after assembly with the other prosthetic components, by dynamic evaluation to obtain a comfortable and functional prosthesis.

The socket casting process just described is currently the most widely used in clinical practice. It reflects that over the last few decades, prosthetists have developed various techniques for both casting and positive shape rectification that produce well-fitting prosthetic sockets able to provide comfort, protect soft tissue, and allow effective control of the prosthesis [2,3]. Design approaches include the quadrilateral, ischial containment (IC), and subischial (SI) sockets [2,12]. There are also variants of the IC such as the Contour Adducted Trochanteric-Controlled Alignment Method (CAT-CAM) [13] and the Marlo Anatomical Socket (MAS) – an ischial-ramal containment (IRC) socket [14,15]. These approaches primarily differ in the management of the proximal socket region (i.e., brim), which is considered critical for stability, load transfer, and comfort. In the quadrilateral design, a narrow anterior-posterior dimension helps to keep the ischial tuberosity seated on a horizontal posterior shelf. A wider medio-lateral dimension allows proximal soft tissue to enter the socket but sometimes at the expense of coronal support [16]. Issues with medio-lateral stability led to the IC design and its variants wherein the ischial tuberosity and part of the ischial ramus are enclosed within the socket to enhance medio-lateral stability and control coronal plane femoral alignment. In the IRC-MAS design, the ischial ramus is enclosed more medially, allowing for the posterior and anterior sections of the proximal brim to be lowered, thus increasing hip motion and socket comfort [15]. These designs require precise shaping of the containment region and appropriate distribution of pressure over load-tolerant anatomical areas. Across these designs, various proximal dimensions (e.g., medial anterior-posterior, skeletal medio-lateral, diagonal medio-lateral, and ramus angle) are critical for appropriately designing the proximal region and are considered key determinants of socket fit and performance. These approaches also rely on global volume adjustments of the thigh tissue for some load transfer [17]. By comparison, subischial (SI) sockets have much lower proximal brims that aim to avoid contacting the pelvis with a similar goal of improving hip mobility and comfort [18,19]. In the SI design load transfer occurs exclusively through thigh tissue, but the proximal medio-lateral dimension of the socket and contouring posterior-lateral to the femur (similar to the CAT-CAM socket [13]) remain important criteria for socket stability [11]. The exact choice of socket, and whether it explicitly follows a particular design, is hybridized or further customized, is based on many factors including the patient’s residual limb, their daily activities, and the prosthetist’s expertise. Hence, transfemoral socket design is considered a complex process with the contribution of different parameters to the assessment of “socket fit” dependent on the specific socket design approach and patient presentation.

Unfortunately, it is difficult to make the processes associated with socket fabrication repeatable and reproducible within and between prosthetists because they are based on implicit knowledge [9,10,20]. This is one reason why, since the 1980s, the prosthetic field has moved toward digitization of design and manufacturing processes [6,20,21]: first by introducing scanning techniques that digitally capture the residual limb/UP/RP/socket shapes, allowing for digital storage and visualization; then by introducing computer-aided design (CAD) software digital rectification templates [22–30], and computer-aided manufacturing (CAM), to digitally rectify the meshes [11,31]; and finally, by fabricating the socket with new materials and techniques (e.g., 3D printing) [32–37].

These innovations (3D scanners, CAD-CAM technologies, new materials and techniques) have benefitted prosthesis users, prosthetists, and clinics [20], by reducing fabrication time, physical storage space, and exposure of prosthetists to unhealthy biomechanical factors and irritants, and by allowing for documentation of fabrication steps [6,10,11,31,36,38–42]. Despite these benefits, current techniques rely on the prosthetist’s ability to make CAD rectifications or to adapt a predefined rectification template to an individual’s unique residual limb characteristics [20].

More recent advancements have explored Artificial Intelligence (AI) algorithms. For example, digital tools such as Instalimb (Sumida-ku, Tokyo, Japan) use AI to recognise anatomical landmarks on coloured scans of the residual limb speeding-up the rectification process [43]. In other examples, researchers trained AI (e.g., Principal Component Analysis, K-means, DiffusionNet) to capture the knowledge used by prosthetists when completing rectifications by analysing differences between the digital meshes captured before and after rectification [20,22,44–49], relying mostly on geometric parameters. These approaches capture both the typical rectifications adopted by prosthetists on whom the AI was trained, but also the variations used to adjust the typical rectification for individuals with amputation. This knowledge has two components: “explicit” and “implicit” [20], with prosthetists able to verbally describe the first but not the second. Importantly, the proposed approach to AI algorithms can be trained on a pool of prosthetists resulting in the acquisition of “shared knowledge” [22,44–46,48] or on a single prosthetist to capture their specific rectification method [20]. The result of this AI learning process is a new type of “AI rectification template” that assists a prosthetist in preparing a socket for a new individual with amputation, by applying the typical learned template and its variations, while also preserving their location, magnitude, and synergistic relationships.

All AI algorithms proposed to date have focused on the rectification of transtibial sockets [20–22,44–46,50–52], with only two exceptions for transradial sockets [47,49]. No AI-driven algorithms have been reported yet for people with transfemoral amputation, despite the provision of these sockets being challenging due to the mass of soft tissue surrounding the bone. To tackle this gap in knowledge, this proof-of-concept work aimed to demonstrate the feasibility of defining a prosthetist-specific AI rectification template to support the provision of transfemoral sockets, leveraging the workflow described in Cutti et al. [20], and to test its validity using a leave-one-out approach.

## Materials & methods

### Participant characteristics

This work constitutes retrospective analysis of existing data. Data from nine individuals with transfemoral amputation who participated in a larger, multisite clinical trial (NCT04141748) [20] were accessed for this study on November, 10, 2025. The study from which data were drawn was approved by the Institutional Review Boards of the primary institutions (STU00210416; VAM-19–00466; 714–2019-SPERAUSLBO-19134), as well as the Department of Defense Office of Human Research Oversight (E01091). The original clinical trial recruitment took place between September 1, 2020, and ended on September 1, 2023, and all participants gave written informed consent to participate in the original clinical trial. All authors were investigators in the original clinical trial and, as such, had access to information that could identify the individual participants during conduct of the original clinical trial. The subset of data that is the focus of this analysis came from a prosthetist with 20 years of experience in fitting sockets to people with transfemoral amputation. Table 1 reports the participant demographics (sex, age, mass, height, aetiology of amputation) and residual limb characteristics (side, length, and tissue type).

**Table 1:** Characteristics of the participants with transfemoral amputation included in the study. ID: identifier; M: male; F: female; BMI: body mass index; R: right; L: left.; SD: Standard deviation.

ID	Sex (M/F)	Age (years)	Mass (kg)	Height (cm)	BMI	Side (L/R)	Aetiology	Residual Limb Length (cm)	Residual Limb Tissue Type
1	F	33	52	159	20.6	R	Trauma	23.0	Soft
2	M	22	59	175	19.3	R	Trauma	25.0	Medium
3	M	44	94	178	29.7	R	Trauma	29.5	Firm
4	M	27	82	180	25.3	L	Trauma	25.0	Firm
5	F	19	40	155	16.6	L	Trauma	20.0	Medium
6	M	53	85	178	26.8	R	Trauma	26.5	Medium
7	M	19	69	183	20.6	L	Trauma	33.5	Medium
8	M	47	77	182	23.2	L	Trauma	32.0	Medium
9	M	46	68	172	23.0	L	Trauma	26.5	Medium
Mean ± SD	F (22%) M (78%)	34 ± 13	70 ± 17	174 ± 10	22.8 ± 4.3	L (56%) R (44%)	Trauma (100%)	26.8 ± 4.3	Soft (11%) Medium (67%) Firm (22%)

### Process for generating a prosthetist-specific rectification template

To build the prosthetist-specific AI rectification template we performed the following steps: (1) UP manual casting; (2) UP digital 3D scanning; (3) digital identification of landmarks on the UP (in short “UP virtual landmarking”); (4) RP manual fabrication; (5) RP digital 3D scanning; and (6) unsupervised training of the AI algorithm. These steps are summarized below, but detailed information can be found in Cutti et al. [20]. The only exception is *Step 3,* which is unique to application of this process for transfemoral sockets.

**Fig 1:**
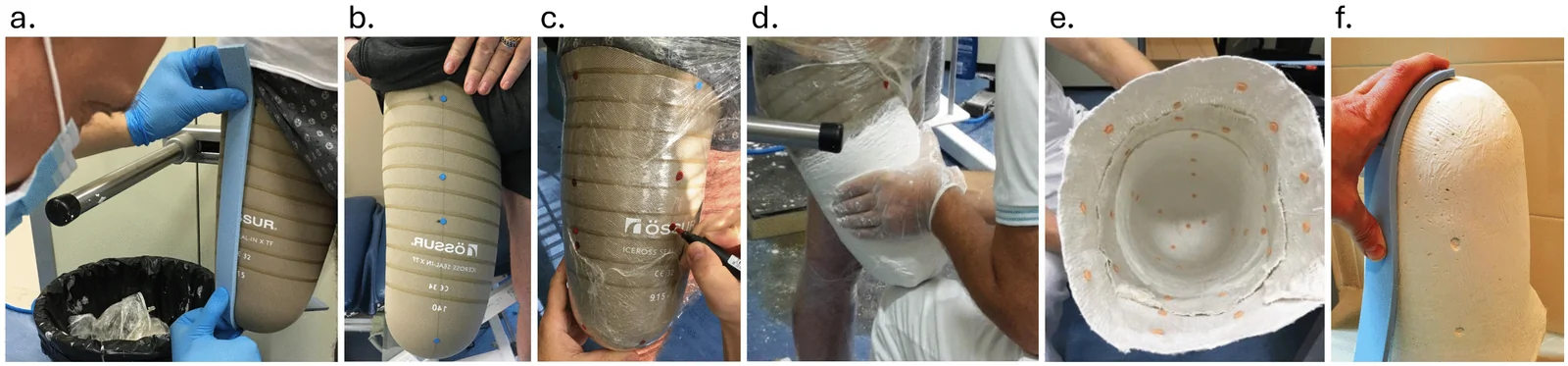
Unrectified positive manual casting. Step 1 of manual casting. a-c: identify landmarks (LMs) on the participant’s residual limb and mark them on the liner; d: hand casting with wet plaster bandages during which the ink LMs transfer to the negative cast; e: internal view of the negative cast extended proximally using a plaster ring, with physical markers glued over the ink LMs; f: plaster positive exhibiting indentations from the physical markers.

**Fig 2:**
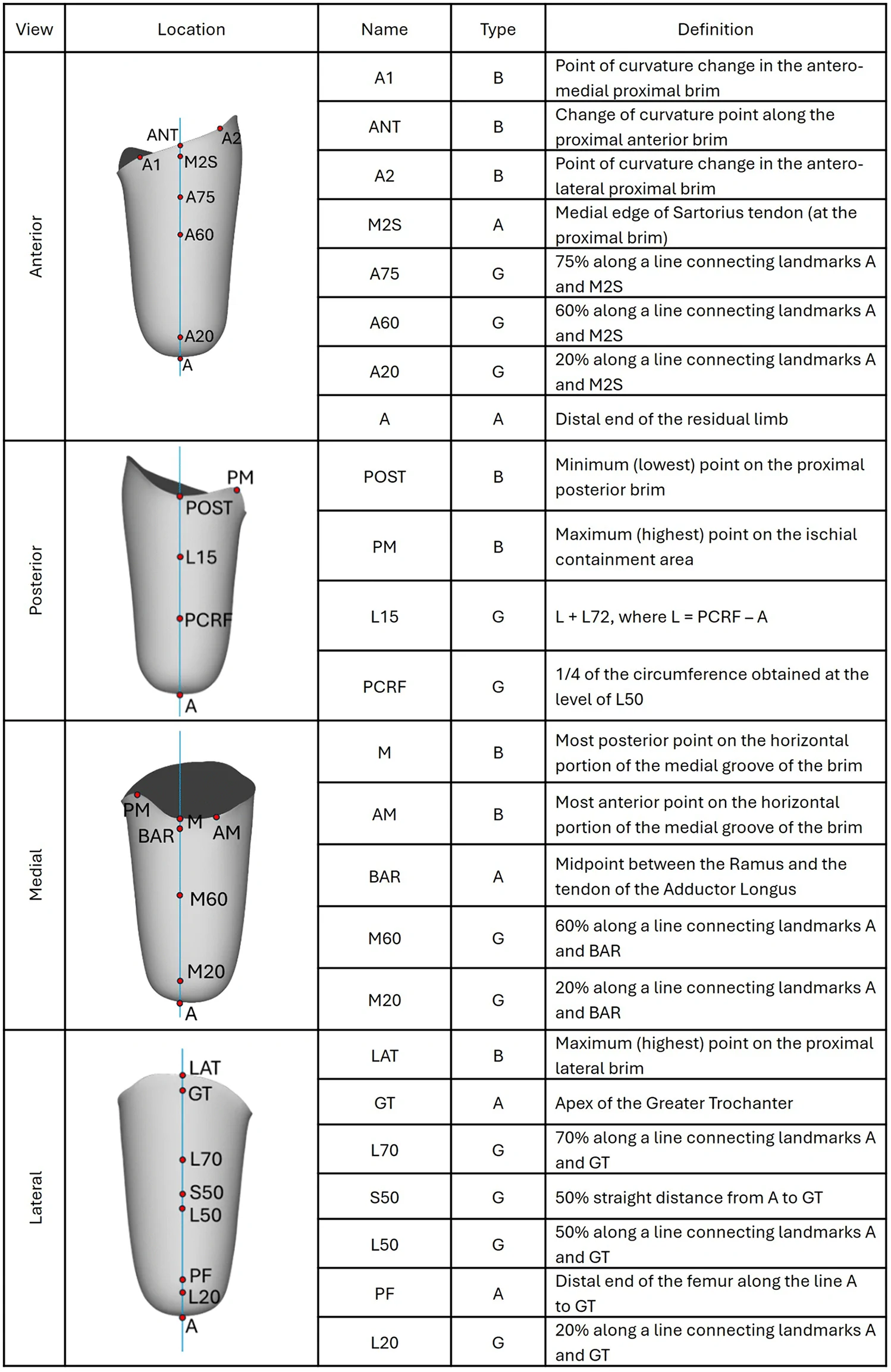
Identification of landmarks. Landmarks identified and marked by the prosthetist on participants’ liner-clad residual limb or directly on the digital scan of the unrectified positive (UP). Abbreviations: A – anatomical location; G – geometrical location; B – locations along the proximal brim.

In *Step 1*, the prosthetist manually fabricates the UP by taking a plaster cast of each participant’s residual limb over a liner (Seal-In X, Ossur, Reykjavík, Iceland), with the subject standing. The key actions taken during this step are illustrated in Fig 1. During casting the prosthetist identified a set of landmarks (LMs) (Fig 2) on the participant’s residual limb, used stickers to identify those LMs on the liner worn over the residual limb, and marked the location of the stickers with a felt pen on the cling film wrapped around the liner (Figs 1a-1b). These ink LMs were transferred to the inner surface of the negative cast by the wet plaster bandages capturing the ink (Figs 1c-1d). Then, the negative cast was extended proximally by 15 cm using plaster bandage wrapped in a ring. A set of about 20 physical markers 7 mm in diameter and 2 mm in thickness were glued to the inside of the cast over the ink landmarks and randomly on the plaster extension (Fig 1e). By filling the negative cast with liquid plaster, the UP was obtained, and all marks were transferred to it as indentations (Fig 1f). Some of the LMs reported in Fig 2 represent anatomical locations identified by palpation (“type A”), while others are geometric locations (“type G”). “Type B” LMs are not used during this stage of the process but are required for, and will be described during, *Step 3*. The prosthetist used a soft ethylene-vinyl-acetate (EVA) strip to mark a line connecting the proximal and distal LMs, and a tape measure to identify the intermediate LMs (Fig 1a).

In *Step 2*, the UP was digitally scanned using the EinScan Pro 2X Plus structured light scanner coupled with the high-definition accessory (Shining3D, Hangzhou, China). Accuracy and repeatability of this scanner for this specific application have been previously reported [53,54].

In *Step 3*, the type “B” LMs described in Fig 2 were virtually identified in the scan. These are geometric LMs that aim to describe the shape of the proximal brim of the socket. Virtual identification was completed by the prosthetist using *SocketFactory*, a custom Python software (version 3.8) that takes advantage of MeshLab (MeshLab_64bit_fp v2020.07) [55] and VTK libraries (version 9.0.0) [56] as computational geometry engines, with Qt (PyQt5 version 5.14.2) [57] to support the graphical user interface [53].

In *Step 4*, the prosthetist manually rectified the UP making sure to leave the marker indentations on the proximal extension of the positive mould untouched. Details about the rectification process are provided in the next section. Once completed, the prosthetist moved on to *Step 5,* digitally scanning the RP using the same process as described in *Step 2*.

**Fig 3:**
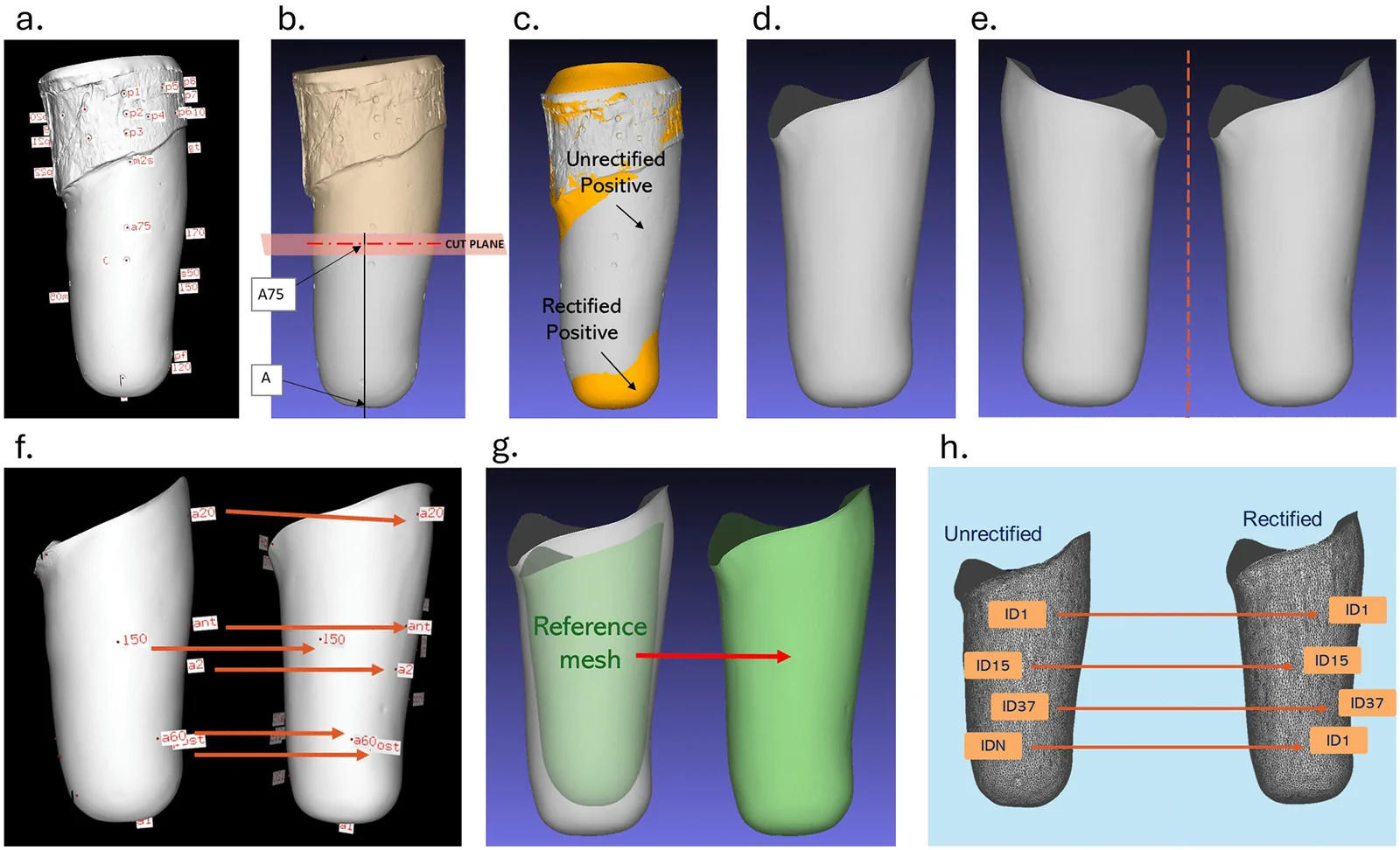
Unsupervised training of the AI algorithm. Processing steps for each Unrectified Positive-Rectified Positive (UP-RP) pair using SocketFactory software. a: digital labelling of the landmarks (LMs) described in Fig 2; b: alignment of the UPs in the virtual global coordinate frame; c: spatial registration of the RPs with respect to the paired UPs; d: digital removal of the proximal extension and cleaning of the meshes; e: mirroring of all right-sided UP-RP couples; f: projection of LMs from the UP to the corresponding RP; g: morphing of UP-RP mesh pairs with a Reference Mesh; h: evaluation of UP*-RP* displacement vectors between homologous vertices.

Finally, in *Step 6*, each pair of UP-RP meshes were digitally processed using *SocketFactory*. As described in Cutti et al. [20] this included: digital labelling of the LMs described in Fig 2 (Fig 3a); alignment of the UPs in the virtual global coordinate frame (Fig 3b); spatial registration of the RPs with respect to the paired UPs using the LMs on the proximal untouched extension (Fig 3c); digital removal of the proximal extension and cleaning the meshes of any imperfections due to the scanning process (Fig 3d); mirroring of all right-sided UP-RP couples (to obtain a dataset of only left-sided meshes) (Fig 3e); projection of LMs from the UP to the corresponding RP (Fig 3f); morphing of UP-RP mesh couples with a Reference Mesh, to obtain a dataset of morphed UP* and RP* meshes with the same topology (Fig 3g) [58]; and evaluation of UP*-RP* displacement vectors between homologous vertices (Fig 3h). Once the dataset was processed, the Principal Component Analysis (PCA) was trained with the dataset of displacement vectors, using the approach described by Dickinson et al. [20,46]. The PCA extracted an average displacement matrix (ADM) and learned and clustered the “modes of variation”, i.e., the synergistic changes the prosthetist applied when adapting their typical rectifications to a specific individual with amputation.

### Manual rectification procedure and check socket fabrication

**Fig 4:**
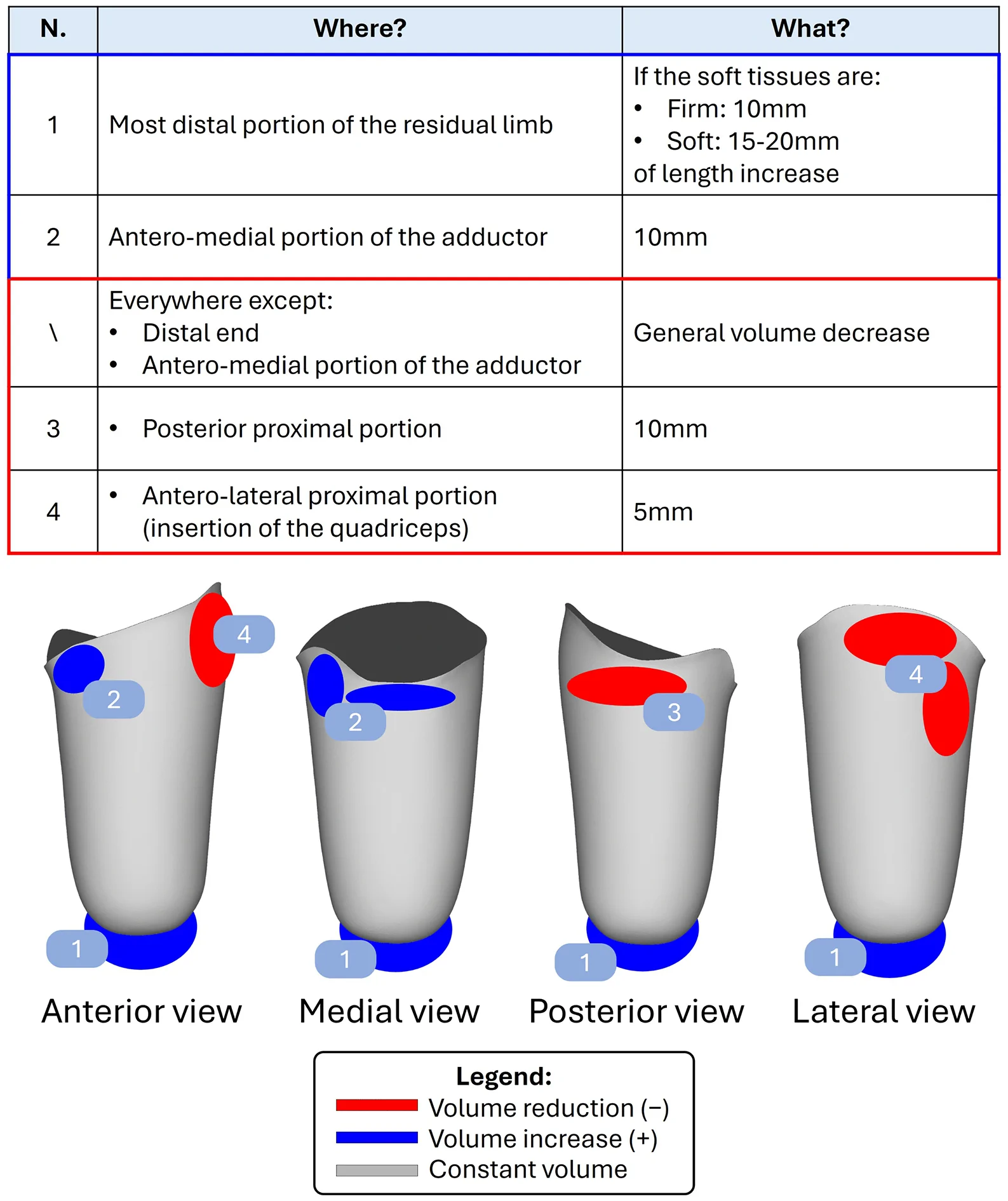
Rectification approach described by prosthetist. Verbal and graphical description of the prosthetist-specific rectification approach. Red shading is associated with a volume decrease, blue shading is associated with a volume increase, and grey shading represents unmodified areas (constant volume).

As illustrated in Figs. 2, 3 and 4, the socket design executed by the prosthetist in this study was a variation of the IC socket with a lower posterior brim inspired by the “extremely low” posterior trimlines of the MAS design [14,59]. The lower posterior brim relieves the gluteus maximus muscle, allowing for freer contraction during hip flexion-extension, without sacrificing containment of the ischial tuberosity or necessitating that containment be moved medially as in the MAS socket. The remainder of the socket geometry follows primarily IC principles and not the proximal dimensions of the MAS socket. The rectification approach as described by the prosthetist is illustrated in Fig 4. First, the distal end of the residual limb is extended by approximately 10 mm for firm tissues and 15–20 mm for soft tissues to accommodate distal tissue deformation under load. An additional 10 mm localized increase is made to the antero-medial adductor region. A general volume reduction is then performed over the entire surface, excluding the distal end and the antero-medial adductor area. Finally, localized reductions are made in the proximal regions: 10 mm in the posterior-proximal area and 5 mm in the antero-lateral proximal area corresponding to the quadriceps insertion.

Using the RP, the prosthetist fabricated a check (i.e., diagnostic) socket consisting of a flexible inner socket made from thermoplastic EVA, placed within a more rigid Polyethylene Terephthalate Glycol (PETG) socket with a U-shaped frame. The check socket was fit to the participant as part of the larger clinical trial [60]. Each participant assessed the socket during static standing and provided the Socket Comfort Score (SCS) [61] and verbal feedback reported in Table 2.

**Table 2:** Socket Comfort Scores (SCS) and verbal feedback provided by participants for the check sockets made by the prosthetist using manual techniques. Std: Standard Deviation.

Subject ID	SCS	Participant comment
1	8	The ischial support is too high.
2	6	The ischial support is too high. Too wide laterally.
3	6	The socket is too wide.
4	6.5	The socket is too wide.
5	9	The distal end of the residual limb does not reach the end of the socket.
6	8	The socket is too tight.
7	5	A sense of pressure at the anterior distal end of the femur. The ischial support is too high.
8	8	None.
9	10	None.
**Mean ± std**	**7.5 ± 1.5**	

### Digital rectification quality assessment

To qualitatively verify the typical rectification rules described by the prosthetist, the average UP* mesh was calculated based on the UP* meshes of the nine participants, and then the ADM was applied to the average UP* to obtain an average RP* mesh. To visually inspect the rectifications embedded in the AI prosthetist-specific rectification template, a distance colour map was generated between the average UP* mesh and the average RP* mesh. Moreover, to assess the synergistic modifications implemented by the prosthetist to account for the specific needs of each participant, the modes of variations learned by the AI algorithm were reported in rank order based on the explained variance. To visually capture the effect of each mode of variation, the average UP* mesh was digitally rectified twice by applying the ADM in combination with that mode, first with a weighting factor of +3 standard deviations and, second, with a weighting factor of −3 standard deviations. Therefore, to illustrate the effect of each mode of variation, two RP* meshes were generated.

### Leave-one-out validation of the AI generated prosthetist-specific template

To validate the AI generated prosthetist-specific template, a leave-one-out approach was adopted. *Steps 1–5* of the procedure previously presented were applied to the whole dataset and then *Step 6* was applied to the processed dataset each time excluding data from one of the participants, overall generating nine slightly different templates, trained with slightly different datasets comprised of eight participants. Hence, nine RP* meshes were obtained.

To quantitatively analyse the results obtained, the RP* meshes were compared with the RP*s made manually by the prosthetist (referred to as “Hand RP”), by calculating ”clinically-relevant measures” [53] consisting of differences in the perimeters of the cross sections and volumes. Agreement between Hand and Digital RP measures is presented using “modified” Bland-Altman plots as introduced by Cutti et al. [53]. Specifically, to ease interpretation of perimeters (often referred to by prosthetists as circumferences) collected at different heights along the residual limb, the heights of the cross sections were reported in percentage on the Y axis of the plot, to resemble the vertical orientation of a residual limb model (instead of the X axis as is typical for Bland–Altman plots) [53]. Since there is no available literature describing the limits within which variation in perimeters or volume of a transfemoral socket will result in a clinically acceptable socket, the results are discussed with respect to the limits formulated by consensus of six prosthetists who participated in the original clinical trial and had clinical experience ranging from 7 to 20 years [60]. The limits for volumes are summarized in Table 3.

**Table 3:** Clinically acceptable limits of variation in socket volume based on the consensus of six prosthetists.

Fit	Increment [%]	Decrement [%]
*Good*	0–3	− 3–0
*Acceptable*	3–8	− 8 to – 3
*Unacceptable*	> 8	<- 8

**Fig 5:**
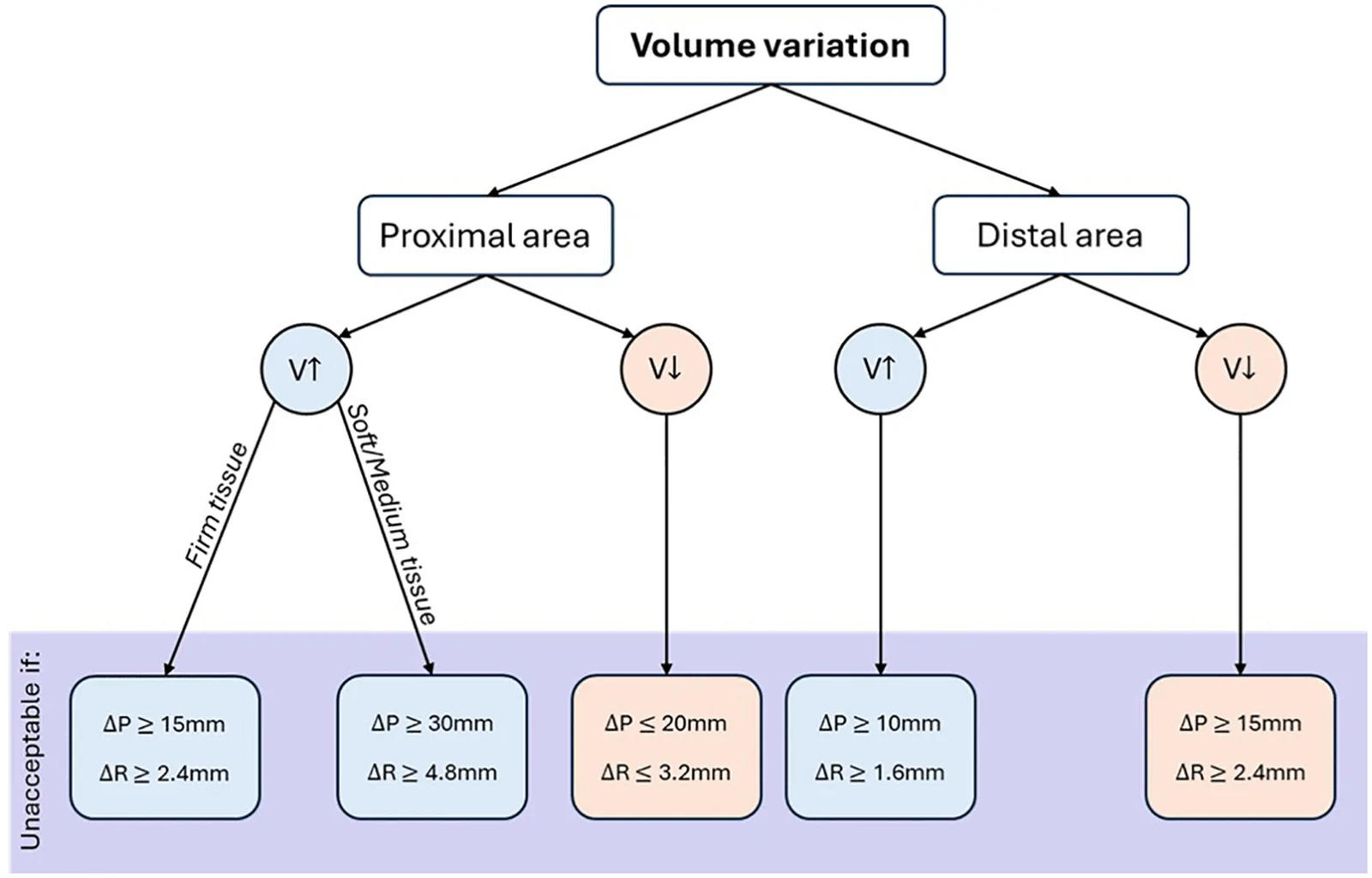
Limits of variation for perimeters. Clinically acceptable limits of variation in perimeters based on the consensus of six prosthetists. V: volume; P: perimeter; R: radius.

The limits of variation for perimeters are reported in Fig 5. Changes in the perimeter of the proximal region of the residual limb are generally more tolerable than those occurring distally. This is because proximal circumferences are typically larger, so a given absolute variation has a proportionally smaller impact compared with the same change in the distal region. Hence, perimeter variations were divided according to whether proximal or distal regions were affected by a volume decrease or increase.

## Results

### Assessment of the rectification rule learned by the AI

**Fig 6:**
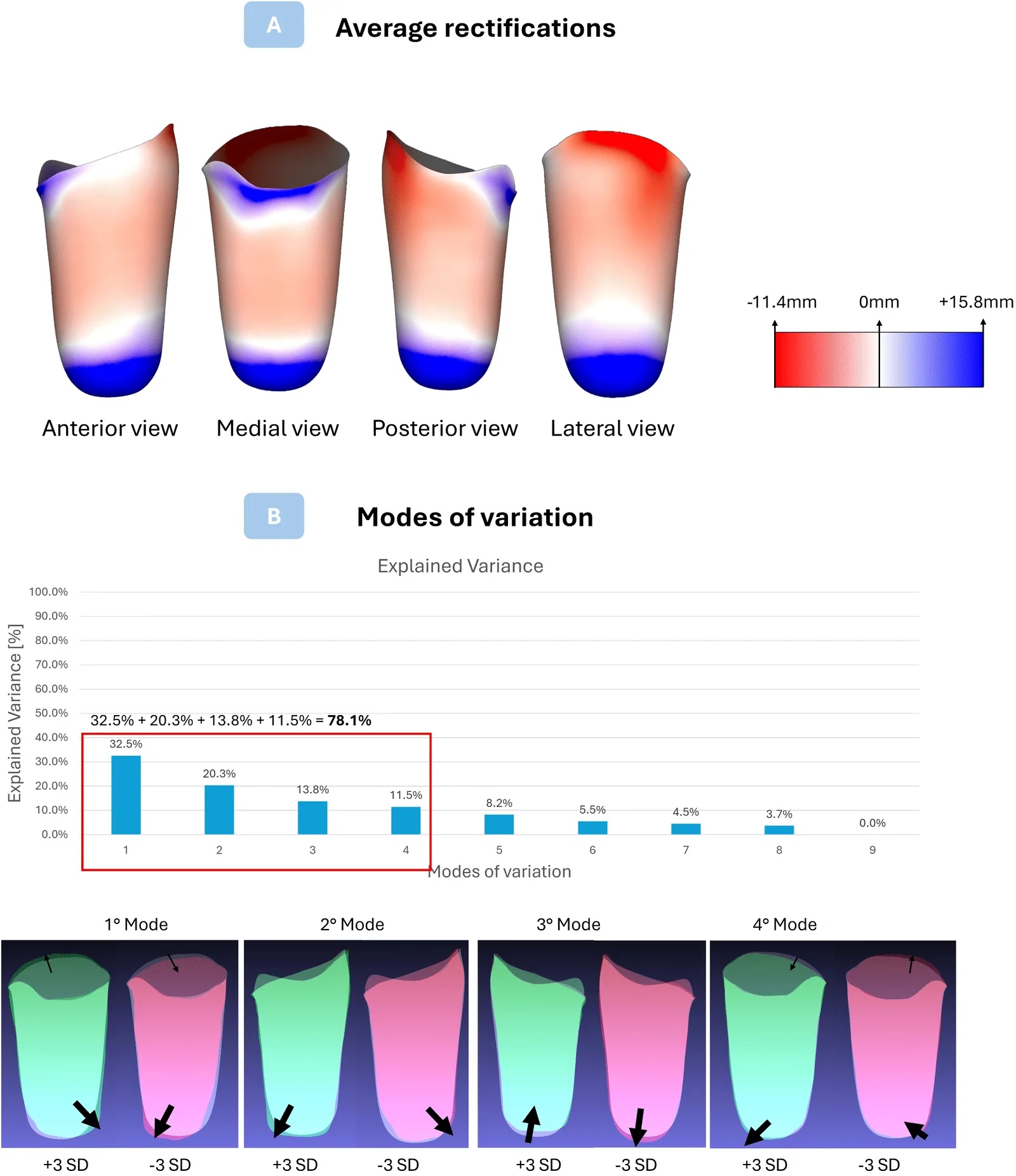
Colour map of the average rectifications. (A) Distance colour maps in the Red–White–Blue scale describing the average rectifications made by the prosthetist during the rectification process. Red was associated with a decrease in material, blue with an increase in material, and white with no change. (B) Plot of the variance explained by each mode of variation and the corresponding qualitative description of the rectifications included in each of the first four modes of variation. Black arrows indicate the location, direction and relative magnitude of the rectifications captured in each mode. SD: Standard deviation.

Fig 6A depicts a colour map of the average rectifications typically performed by the prosthetist. Here, red is associated with material decrease, blue with material increase, and white with no changes. Fig 6B reports the distribution of the explained variance over the modes of variation: specifically, about 78% of variation was explained by modes 1–4. The variance explained by additional modes were less than 10% each. For modes 1–4, Fig 6B also depicts their geometric effect using the + 3 or −3 standard deviation weighting factor. Specifically, the first mode generates a distal anterior (+3 SD) or posterior (−3 SD) widening of the socket; the second mode generates a medial (+3) or lateral (−3) widening; the third mode shortens (+3) or elongates (−3) the distal portion of the socket; and the fourth mode seems related to the creation of reliefs for distal bony prominences or painful spots.

### Digital rectification quality assessment

**Fig 7:**
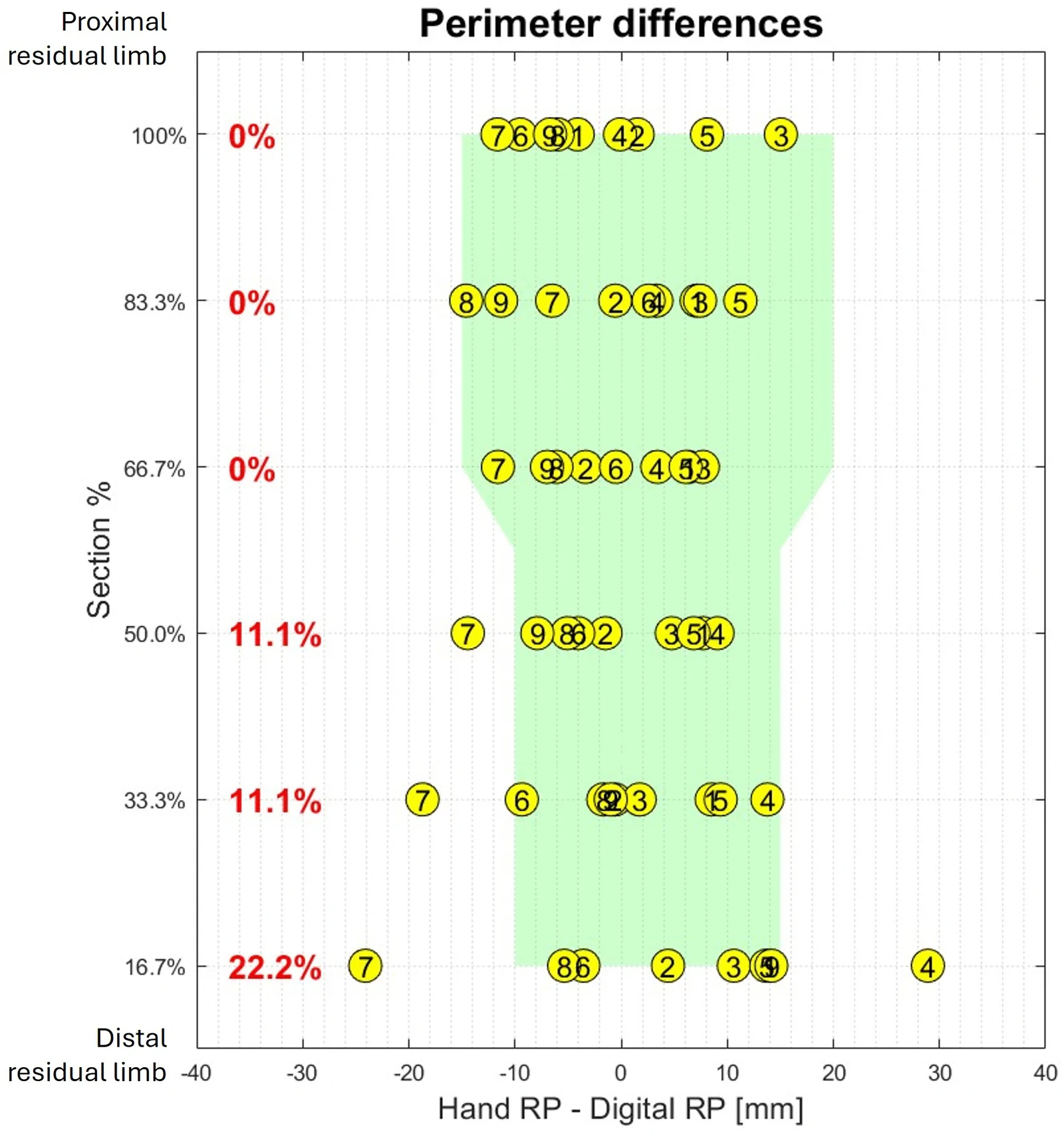
Differences between rectified positives in terms of perimeters. Modified Bland-Altman plot of variation in perimeter differences between RPs at 6 equally spaced cross sections along the longitudinal axis of the residual limb. Each participants’ measurement is reported using their ID inscribed in yellow circles. The green band represents the most stringent limit of variation identified by the prosthetists involved in the larger clinical study [60]. The percentage of unacceptable measures is reported in red for each cross section. Data used to create Fig 7 are provided as S1 File.

To assess the quality of the AI-based rectification through the leave-one-out approach, the clinically-relevant measures are reported in Fig 7. This is a graphical representation of the differences between RPs in terms of perimeters at six equally spaced cross sections along the longitudinal axis of the residual limb. The percentage of unacceptable measures are reported in Fig 7. This was calculated as the percentage of total participants outside the acceptable limit of variation.

Finally, Table 4 reports the volume differences between RPs in absolute and percentage values for each participant. To evaluate the results obtained in terms of percentage volume variations, prosthetists’ opinions are reported in Table 3.

**Table 4:** Volume differences between RPs are reported in absolute and percentage values for each participant. Average values are reported in the last row. Green shading indicates volume differences falling in the “good” fit range, while yellow shading indicates volume differences falling in the “acceptable” fit range.

Subject ID	Volume difference [dl]: DigitalRP−HandRP	Volume percentage difference [%]: $\frac{DigitalRP-HandRP}{HandRP}\%$
**1**	−0.40	1.17
**2**	0.58	−1.92
**3**	1.09	−3.37
**4**	1.10	−3.28
**5**	1.31	−6.39
**6**	0.71	−1.98
**7**	−1.86	4.74
**8**	−1.47	3.75
**9**	−0.70	2.44
**Mean**	**0.04**	**−0.54**

## Discussions

This proof-of-concept study aimed to develop an AI-driven rectification template for transfemoral sockets, leveraging the procedure reported in our previous work on transtibial sockets [20]. The template was based on an unsupervised machine learning algorithm, PCA, which can automatically generate a suggestion for the RP shape based on the UP of a participant with a transfemoral amputation. The current study should be interpreted as a proof-of-concept focused on knowledge capture, not performance optimization of the socket.

To qualitatively evaluate the consistency of the average rectification template learned by the AI algorithm with the rectification technique described by the prosthetist, we compared Fig 4 with Fig 6. This comparison illustrated agreement between the two, i.e., the algorithm correctly identified the areas where the prosthetist typically added or removed material from the UP. Specifically, the UP underwent a general reduction in volume to improve coupling of the socket with the residual limb. Furthermore, material was removed locally from the proximal-lateral and posterior regions. Material was added to the distal end of the positive residual limb mould, creating an extension of approximately 15–20 mm and to the proximal antero-medial portion intended to make the proximal brim more comfortable at the level of the ischial support.

The synergistic modifications that the prosthetist applied to customize the average template to the specific participant (i.e., the implicit knowledge of the prosthetist), were captured by analysing the modes of variation identified by the PCA as described in Fig 6b. The first four modes (which explained 78.1% of the total variance), described rectifications applied at the distal end, which appear to be the areas subjected to participant-specific adaptation of the average template. In detail, the first, second and fourth modes of variation described build-ups either anteriorly/posteriorly (± 3 SD), medially/laterally (± 3 SD), or postero-medial/antero-lateral (± 3 SD), respectively. Finally, the third mode described elongation (−3 SD) or shortening (+3 SD) of the residual limb length. These modifications are likely aimed at creating relief areas for painful spots, or to off-load pressure from the distal end of the residual limb, which is commonly sensitive and painful in those with amputation that transects a long bone such as the femur.

Also, the study aimed to assess the validity of the AI predictions by comparing the Digital RP to the Hand RP, using a leave-one-out approach assessing perimeter and volume differences. Fig 7 shows that, for the proximal region, all participant perimeters fell within limits agreed to by the six prosthetists (see Table 3). However, it also shows that distally, the RP predicted by the AI for Participant 7 was wider than the Hand RP. The opposite occurred for Participant 4, whose Digital RP was less wide than the corresponding Hand RP at the most distal cross section. Interestingly, these outliers are consistent with those who reported low Socket Comfort Scores for the check sockets based on the Hand RP, i.e., 6.5 for Participant 4 and 5 for Participant 7. Additionally, during the fitting process, Participant 7 reported pressure at the anterior end of the distal femur, while Participant 4 reported that the socket was too wide (Table 2). The Digital RPs have a shape that would seem to address these complaints. While we may speculate that the AI-generated sockets may have resulted in better comfort, further studies are needed to confirm this hypothesis. While we believe that AI can assist prosthetists in their decision making and facilitate workflow, its superiority in autonomous rectification is not yet supported and was not the aim of this study. These data simply suggest that further efforts in AI training may consider Socket Comfort Score as an additional outcome measure.

The results reported in Fig 7 for perimeters were mostly acceptable. Similarly, the results reported in Table 4 suggest that for all participants the volume differences between Hand and Digital RPs fall within a “good” or “acceptable fit” range. However, evaluation of the acceptability of our results was based on perimeter and volume thresholds identified through consultation with six experienced prosthetists. This approach was necessary given that no literature was available on this topic for people with transfemoral amputation. As such, we believe this study is the first to propose acceptability thresholds for transfemoral socket fit based on perimeters and volumes. However, these thresholds need additional exploration and validation.

While our results are promising, this study has limitations. First, it should be considered exploratory given that the sample size cannot reflect the extent of variability in the clinical presentation of people with transfemoral amputation. For example, all participants had traumatic amputations. Other common amputation aetiologies, such as vascular disease, were not represented. Second, the study included a single prosthetist providing high intra-prosthetist consistency in terms of the outcome parameters but limiting generalizability of the approach to rectification. Future studies are needed to demonstrate that this same approach can be used effectively across many prosthetists and rectification patterns intended for different socket designs. Third, the workflow required identification of an extensive number of landmarks. We chose intentionally to have high redundancy in this first attempt. Future studies may perform sensitivity analyses to determine the minimum number of landmarks required to generate models with acceptable performance that may be more efficient to use in clinical practice. Currently, the location of Type B landmarks is specific to the brim shape of the socket used in this study and may need to be redefined depending on the socket design. Additionally, based on the clinical trial protocol from which these data were drawn [60], socket comfort was evaluated only under static conditions. Socket comfort under dynamic conditions would be required to obtain a more comprehensive understanding of overall socket performance.

Finally, results rely primarily on geometric agreement (perimeter and volume differences), which may not fully reflect clinical socket performance, particularly in transfemoral populations where pressure distribution and stability are critical. Other participant characteristics (e.g., residual limb tissue consistency, scars, position of neuromas, painful areas, volume fluctuation) and the inclusion of patient-centered outcomes regarding the quality of fit should be considered in future studies to design a multiparametric training dataset that, together with geometric parameters, could improve AI predictions. Further clinical parameters may also be included describing the quality of socket fit, but these might depend on the specific socket design. For instance, for an IC socket, prosthetists may need to evaluate if the adductor longus tendon is properly positioned in the medial-anterior aspect of the socket without excessive pressure, or whether the anterior-medial wall allows the pubic ramus to exit without discomfort, or whether there is any gapping or localized pressure at the trim lines. Additionally, it is important to verify that no excessive pressure is experienced with increased weight-bearing, that the socket maintains appropriate tightness under lateral forces, and that key anatomical landmarks, such as the greater trochanter, remain comfortable.

## Conclusions

This proof-of-concept study illustrates application of an AI algorithm to transfemoral prosthetic sockets. We demonstrated that the algorithm could learn the rectifications typically made by a single prosthetist. A key factor in the success of this method was the introduction of a novel, step-by-step approach to data collection and processing, combined with a “morphing” stage that ensured consistent topology between UP and RP mould pairs. Once optimized, this technique could serve as a valuable time-saving resource for prosthetists by automating rectifications that result in a well-fitting initial socket for individuals with transfemoral amputation. This approach has a wide range of potential applications, from supporting digital rectification, to the creation of average rectification maps as a teaching aid. This may be particularly beneficial in resource-limited settings and isolated communities where experienced prosthetists are scarce.

## Supporting information

S1 File Data used to creat Fig 7. (XLSX)

## Abbreviations

UP: unrectified positive
RP: rectified positive
CAD: computer-aided design
CAM: computer-aided manufacturing
AI: artificial intelligence
SCS: Socket Comfort Score
LM: landmark
PCA: Principal Component Analysis
ADM: Average Displacement Matrix
IC: Ischial Containment
IRC: Ischial-Ramal Containment
MAS: Marlo Anatomical Socket
SI: Subischial
CAT-CAM: Contoured Adducted Trochanteric-Controlled Alignment Method
SD: Standard Deviation
UP*: Morphed unrectified positive
RP*: Morphed rectified positive
EVA: Ethylene-vinyl-acetate
PETG: Polyethylene Terephthalate Glycol.
